# Cancer Awareness Measure (CAM) and Cancer Awareness Measure MYthical Causes Scale (CAM-MY) scores in Pakistani population

**DOI:** 10.1038/s41598-022-13012-8

**Published:** 2022-05-25

**Authors:** Rimsha Munir, Naila Noureen, Muniba Bashir, Naila Shoaib, Arifa Ashraf, Jan Lisec, Nousheen Zaidi

**Affiliations:** 1 Cancer Biology Lab, Institute of Microbiology and Molecular Genetics, University of the Punjab, Lahore, Pakistan; 2 Hormone Lab, Lahore, Pakistan; 3 Cancer Research Centre, University of the Punjab, Lahore, Pakistan; 4 Department of Analytical Chemistry, Bundesanstalt für Materialforschung und -Prüfung (BAM), Richard-Willstätter-Straße 11, 12489 Berlin, Germany

**Keywords:** Cancer, Health care, Risk factors

## Abstract

Lifestyle modifications could prevent almost one-third to one-half of all cancer cases. The awareness of cancer risk factors could motivate people to make such changes in their behaviors and lifestyles. This work aims to investigate the cancer awareness level in the Pakistani population. Telephone interviews of 657 individuals in Pakistan were carried out using the Cancer Awareness Measure (CAM) and Cancer Awareness Measure–MYthical Causes Scale (CAM-MY). We observed that participants scored significantly better on the CAM scale than the CAM-MY scale, and CAM scores were negatively associated with CAM-MY scores. Years of formal education or a biology major at undergraduate or graduate level did not affect our population's cancer awareness levels. Age displayed a weak but statistically significant negative association with CAM scores. Most participants failed to identify modifiable cancer risk factors, e.g., low physical activity. Efforts should be made to improve awareness of modifiable risk factors. We observed that brief training sessions could markedly improve people's understanding of cancer risk factors and myths.

## Introduction

According to some estimates, lifestyle modifications alone could prevent almost one-third to one-half of all cancer cases^1,2^. Before people comply with such changes, they require a better understanding of lifestyle and environment-related cancer risk-factors. Although awareness alone may not be sufficient to motivate these modifications^3^, it may influence individuals' lifestyle and behavior. Data from multiple European countries indicate a generally low awareness level on the environment and lifestyle-related causes of cancer in different populations^4^. Previous studies show that cancer awareness level is associated with socioeconomic status, ethnicity, and education levels^4^. These studies also observed that most people recognize the prominent causes of cancer, such as smoking. In contrast, other lifestyle-related risk factors such as obesity and low fruit and vegetable intake are often poorly recognized^4^.

People often associate certain lifestyle and environment-related items, which do not significantly impact cancer development, with increased cancer risk. Cell phone usage or consumption of genetically modified food would be examples of such mythical causes of cancer. Previous works have also studied the prevalence of such beliefs in the general population^5,6^. These fallacies misdirect people, and instead of bringing about positive changes in their lifestyle that could reduce their cancer risk, they focus on minimizing risk via confronting such mythical risk-factors. In the past, the tobacco industry has also deliberately funded work on spurious risk factors for neoplastic and cardiovascular diseases to distract people from one of the major risk factors, i.e., smoking^7^.

The studies mentioned above are mostly conducted in developed countries where the literacy rate is generally higher than in underdeveloped countries. To the best of our knowledge, none of the previous studies have performed an extensive analysis of the prevalence of beliefs about actual or mythical causes of cancer in the Pakistani population. The presented work aims to study the level of cancer awareness in this population. We will also evaluate the impact of various socio-demographic factors on the awareness level.

## Methods

### Ethics approval and consent to participate

This study, questionnaires and consent form were reviewed and approved by Cancer Research Centre Bioethics Committee, University of the Punjab, Lahore, Pakistan, approval number is D/592/CRC. Written informed consent was obtained from the participants with WMA Declaration of Helsinki-Ethics principles for research involving human subjects.

### Assessment of awareness on cancer causes

**Figure 1:**
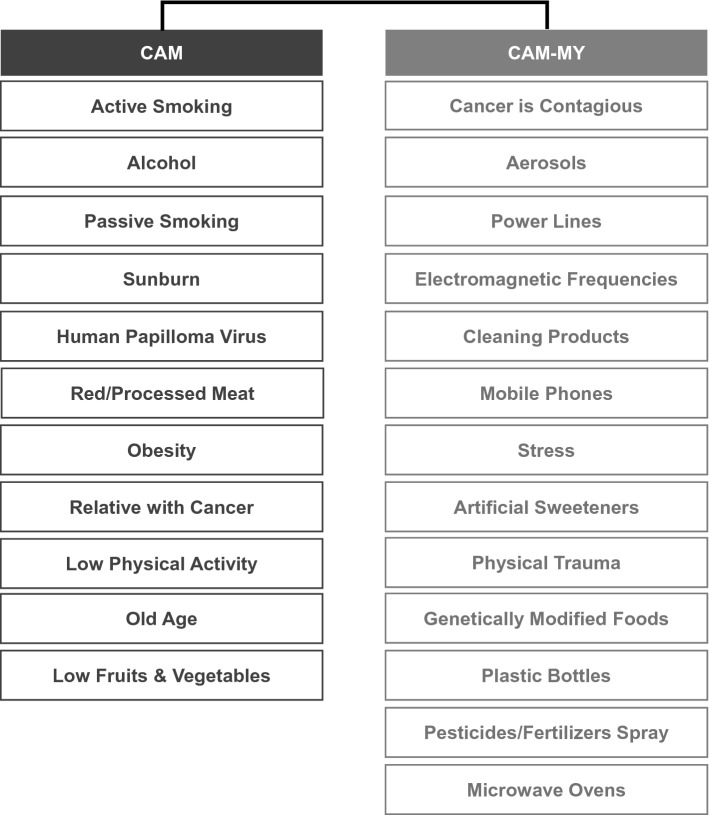
CAM and CAM-MY items.

CAM is the most frequently used and validated tool for assessing awareness of known cancer risk factors^8^. More recently, Smith et al. developed a new tool to assess people's belief in common cancer-related myths called the (CAM-MY)^4^. This scale has been developed and validated on the UK population. Here, we conducted a pre-survey to assess whether some other myths are more prevalent in the Pakistani population. For this pre-survey, 1000 randomly selected participants were approached and asked about the major causes or risk-factors of cancer. Most of the misconceptions about cancer were similar to what has already been identified by Smith et al.^4^. Two other myths prevalent in our population were “cancer is a contagious disease” and “pesticides, or fertilizers spray cause cancer.” Hence, these items were also included in the questionnaire. The items selected for CAM and CAM-MY scales are displayed in Fig. 1. There were 11 CAM, and 13 CAM-MY items. In the initial stages of the project, a team of multidisciplinary researchers reviewed these measures to assess their applicability for the Pakistani population. The items included in these measures were translated into local languages, and the terminology was made more applicable to the Pakistani setting. Data on CAM and CAM-MYCS were collected via telephonic interviews. Informed consent was obtained from each participant before the interview. The participants were presented with CAM and CAM-MY items in a mixed manner, and they were asked whether they considered these as a risk factor for cancer. The response values r were recorded on the five-point (5 to 1) Likert scale, i.e., Strongly Agree, Agree, Neither Agree Nor Disagree, Disagree, Strongly Disagree. Strongly Agree received the highest score on the CAM scale i.e. 5, and Strongly Disagree received the lowest score of 1. Before calculating CAM and CAM-MY scores using formula, response values for CAM-MY items were inversed by – 1 × (r − 6). Thereby, we ensure that both final scores, CAM and CAM-MY are high for correct answers, i.e. when participants correctly agree with CAM items and correctly disagree with CAM-MY items. Both scores were calculated using the responses to the respective items by (sum(r)-n)/4 n where r is the vector of responses, each containing a value between 1 and 5, n is the total number of questions under consideration and sum(r) is the total number of points given in n responses.

To compare the cancer risk factor perception of medical professionals against a control group, cancer experts (oncologists and medical doctors, n = 25) were recruited through professional networks, whereas non-experts (exclusion criteria: medical professionals, cancer researchers, individuals that formally studied cancer-related subjects, e.g., oncology, cancer genetics/biology at undergraduate or graduate levels, n = 25) were randomly selected from the general population.

To assess the effect of training sessions on CAM and CAM-MY scores, we recruited 40 students from University of the Punjab studying non-biological subjects. These participants were presented with the questions, including CAM and CAM-MY items at baseline levels. After completing the baseline interview, the sample was randomized 1:1 to either the intervention or control group. The intervention group attended a brief training session (20 min) at CBL-MMG, University of the Punjab, describing general information regarding cancer development, the link between cancer and lifestyle behaviors, and commonly held myths about cancer causes. These training sessions were conducted by an expert (Ph.D. in Molecular Genetics) specializing in Cancer Biology. In these sessions, the participants were provided information in their local language (Urdu) on cancer myths and facts (CAM and CAM-MY items). The content for these sessions was derived from the official website of the National Cancer Institute: https://www.cancer.gov/about-cancer/causes-prevention/risk, https://www.cancer.gov/about-cancer/causes-prevention/risk/myths. The control group did not receive any intervention. After 48 h, the intervention and control group participants were presented with the same questions, and their scores were recorded.

### Study-design and participants

A total of 657 participants (inclusion criteria: participants aged ≥ 16 years, minimum qualification ten years of formal education, able to speak and understand English or Urdu) took part in this cross-sectional study. In order to obtain the necessary personal information, each participant completed a structured questionnaire. The STROBE guidelines were followed. We used cohort, case–control, and cross-sectional studies (combined).

### Health behaviour characteristics

Health behavior characteristics were quantified, as described by Shahab et al.^9^. Briefly, smoking status was assessed by asking participants if they smoked, and respondents were classified as smokers, ex-smokers, or never-smokers. Physical activity levels were also assessed. Participants that claim to engage in physical activity for ≥ 30 min for at least five days a week were classified as meeting current physical activity guidelines^10^. Body Mass Index (BMI) was calculated using the participants' self-reported weight and height, and the participants were classified into two BMI-based categories; < 25 k/m^2^ or ≥ 25 kg/m^2^ (people with BMI of ≥ 25 kg/m^2^ are categorized as overweight). Self-reported fruit and vegetable consumption levels of the participants were also assessed based on^9^. Respondents consuming at least five portions per day were classified as meeting current guidelines.

Aggregated behavior score was calculated as described earlier^9^—scoring each health behavior that did not meet guidelines or indicated greater risk as 1, except for smoking status where current smoking was scored as 2 and past smoking as 1. We did not record the participant's alcohol consumption levels as it is illegal to consume alcohol in Pakistan. Therefore, people tend to lie or hide facts in response to this question. This resulted in an overall health behaviour risk score ranging from 0 to 5, with higher scores indicating greater risk.

### Statistical analysis

The differences between groups were analyzed by ANOVA or *t*-test (paired or unpaired), where applicable. Statistical analyses and graphical representations were performed using the GraphPad Prism, Microsoft Excel, or R software environment 3.4.2 (http://cran.r-project.org/). *P*-values < 0.05 were considered statistically significant and indicated when different.

## Results

### Overall CAM and CAM-MY scores

**Figure 2:**
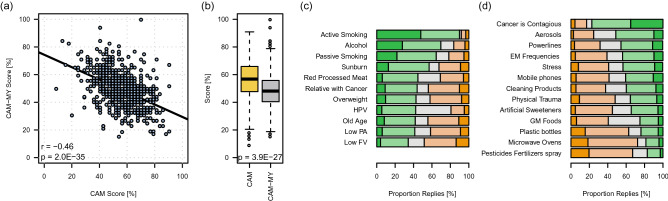
CAM and CAM-MYCS scores in the study-population. (**a**) Correlation between percentage CAM scores and CAM-MY score. Pearson's correlation coefficient (r) and p value are shown (**b**) Overall CAM and CAM-MY scores of the study-participants (n = 657) were compared. The results show the distribution CAM and CAM-MY scores; with the box indicating the 25th–75th percentiles, with the mean indicated line. The whiskers show the range. Significance was determined by *paired t-test* (*P* < 0.0001****). Proportion Replies (%) for each (**c**) CAM and (**d**) CAM-MY questions. The responses were recorded on Likert scale -Strongly agree, Agree, Neither agree or Disagree, Disagree, Strongly disagree. The percentage of respondents that gave correct responses (i.e. strongly agree for CAM items and strongly disagree for CAM-MY items) is presented as dark-green stacks, while the percentage of respondents that gave incorrect responses (i.e. Strongly Disagree for CAM items and Strongly Agree for CAM-MY items) is stacked dark orange. The lighter shades of green and orange show the responses closer to being correct and incorrect, respectively. The grey-shade depicts the percentage of respondents who were neutral (Neither agree nor Disagree).

First, we calculated the correlation between CAM and CAM-MY scores for the overall population. There was a statistically significant strong negative correlation (r =  − 0.46, *P* < 0.0001) between the two scores, which means that better performance on one scale was associated with worse performance on the other (Fig. 2a). We observed that the percent CAM scores (55%) were significantly higher than the percent CAM-MY scores (46%) for the overall study population (Fig. 2b).

Next, we determined which of the CAM and CAM-MY questions were most correctly answered by the participants. We recorded the responses for both measures on the Likert scale (strongly agree, agree, neither agree nor disagree, disagree, strongly disagree). In Fig. 2c and d, stacked-bar graphs display the percent proportion replies for the CAM and CAM-MY scores. The percent proportion of the respondents that gave the most appropriate response (i.e., strongly agree for CAM items and strongly disagree for CAM-MY items) is presented as dark-green stacks. In contrast, the percentage of respondents that gave incorrect responses (i.e., strongly disagree for CAM items and strongly agree for CAM-MY items) is stacked dark orange. The lighter shades of green and orange show the responses closer to being correct and incorrect, respectively. The grey-shade depicts the percentage of neutral respondents (neither agree nor disagree). The most commonly identified risk-factors (CAM items) were active-smoking, alcohol, and passive smoking. The lowest number of correct responses was received for low fruit and vegetable (Low FV) consumption, low physical activity (Low PA), and old age (Fig. 2c, Supplementary Table 1). Moreover, among the CAM items, respondents were most uncertain about HPV, with > 38% percent of the responses being “neither agree nor disagree”.

The most correctly rejected CAM-MY item was “cancer is contagious,” followed by aerosol and powerlines being cancer causes (Fig. 2d, Supplementary Table 2). However, even for the “cancer is contagious” item, we received only ~ 35% most appropriate responses (i.e., strongly disagree) and ~ 42% closer to the correct answers (i.e., disagree). Moreover, the most incorrect responses were received for fertilizer/pesticide spray, microwave ovens, and plastic bottles. Overall, for all the CAM-MY items except for “cancer is contagious,” the percentage of most correct responses remained ≤ 11%. A higher proportion of the participants responded with either agree or disagree for each of the CAM-MY items. They avoided giving more certain answers, i.e., “strongly agree” or “strongly disagree.” Moreover, among the CAM-MY items, respondents were most uncertain about genetically modified (GM) food, with ~ 35% percent of the responses being “neither agree nor disagree”.

### Effect of different socio-demographic factors on CAM and CAM-MY scores

**Figure 3:**
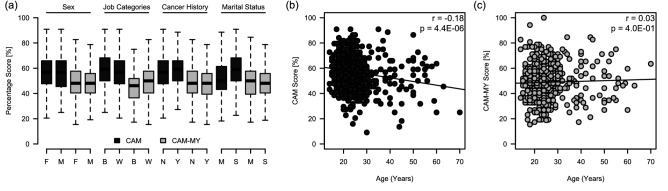
Effect of socio-demographic factors on CAM and CAM-MY scores. (**a**) CAM and CAM-MY scores were compared between: male versus female participants, blue- versus white-collar participants, participants that had versus did not have cancer history (family member or friend with cancer), and single versus married participants. The results show the distribution of CAM and CAM-MY scores; with the box indicating the 25th–75th percentiles, with the mean indicated line. The whiskers show the range. Significance was determined by *unpaired t-test*. Correlation between age and (**b**) CAM scores or (**c**) CAM-MY scores. Pearson's correlation coefficient (r) and *p* value are shown.

We also determined the impact of certain socio-demographic factors on CAM and CAM-MY scores (Fig. 3a). It was observed that both of these measures were not affected by sex, and there was no significant difference between average CAM and CAM-MY scores of male and female participants (Fig. 3a). The scores were also not significantly different between the participants categorized according to their job categories (blue-collar vs. white-collar workers). We hypothesized that a cancer history—having a family member or friend with cancer—may affect CAM or CAM-MY scores. However, we did not observe any difference between these scores for participants with or without a family history of cancer. Marital status, however, affected the CAM scores, and the single participants had significantly higher CAM scores. We hypothesized that this difference could be attributed to the participants' age, as married participants were mostly older. However, we observed that age displayed only a weak but significant negative association (r =  − 0.18, *P* < 0.0001) with CAM scores (Fig. 3b). However, no significant association was noted between CAM-MY scores and the participants' age (Fig. 3c).

### Effect of health behavior characteristics on CAM and CAM-MY scores

**Figure 4:**
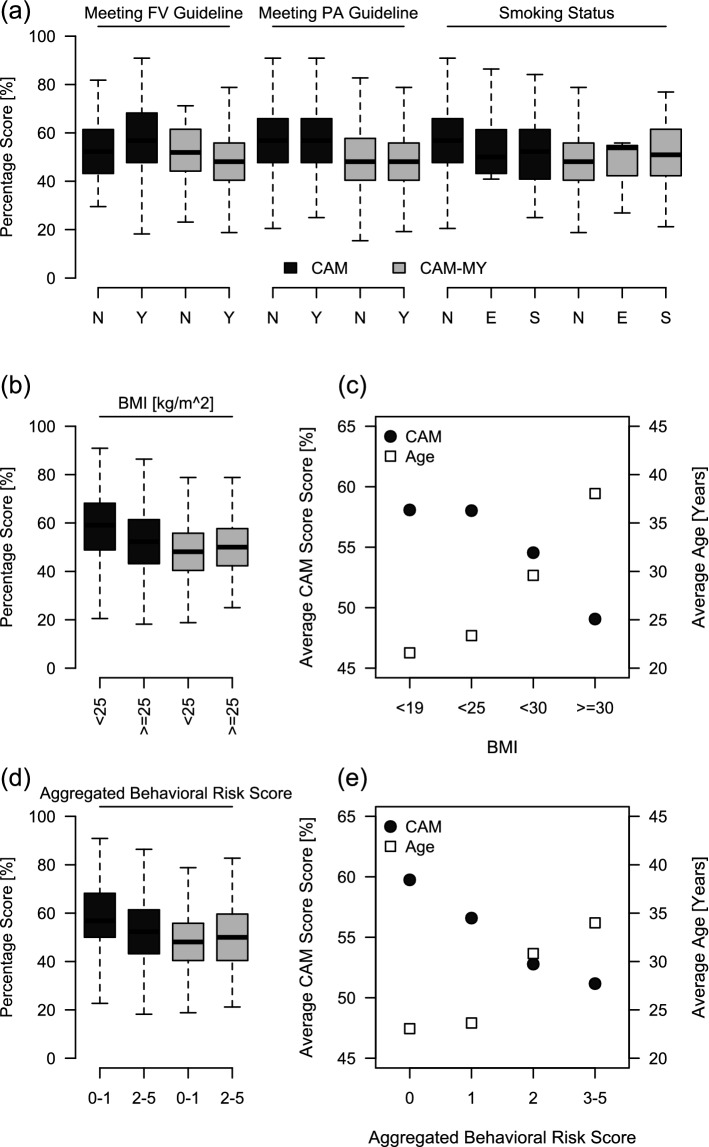
Effect of health behavior characteristics on CAM and CAM-MY scores. (**a**) CAM and CAM-MY scores were compared between the participants meeting *vs.* not meeting the current guidelines of fruit/vegetable consumption (i.e. ≥ 5 portions/day), meeting *vs.* not meeting the physical activity guidelines (i.e. ≥ 150 min of exercise/week) and among the participants with different smoking status (i.e. Smokers, Ex-Smokers or Never Smokers). (**b**) Comparison of CAM and CAM-MY scores between the participants with BMI < 25 kg/m^2^ or ≥ 25 kg/m^2^. (**c**) Average CAM Scores (*left y-axis, yellow circles*) and average age (*right y-axis, purple squares*) of participants in groups categorized according BMI (*x-axis*). (**d**) Comparison of CAM and CAM-MY scores between the participants with aggregated behaviour risk score of 0–1 or 2–5. (**e**) Average CAM Scores (*left y-axis, yellow circles*) and average age (*right y-axis, purple squares*) of participants in groups categorized according to their aggregated behaviour risk score (0, 1, 2 or 3–5) (*x-axis*). The *box-whisker plots* show the distribution of CAM and CAM-MY scores; with the box indicating the 25th–75th percentiles, with the mean indicated line. The whiskers show the range. Significance was determined by Significance was determined by *unpaired t-test or ordinary one-way ANOVA* with Tukey's post hoc analysis.

Shahab et al.^9^ have reported that CAM and CAM-MY scores may also vary among individuals with differential health behavior characteristics, including their fruit and vegetable consumption level, physical activity level, smoking status, and body-weight. Here, we also compared CAM and CAM-MY scores among the groups of participants categorized based on these characteristics (see methods for details) and found no significant difference (Fig. 4a), except for the BMI categories where participants with BMI ≥ 25 scored significantly lower on the CAM scale (Fig. 4b). For our population, age was directly associated with BMI. In the previous section, we showed that age is also associated with CAM scores. Hence, we hypothesized that age might be the driving force contributing to the differences in the CAM scores. To explore that further, we compared the age and CAM scores among the participants categorized based on their BMI. As expected, we observed that with increasing BMI, participants' age increased, while there was an apparent decline in their CAM scores (Fig. 4c).

We also calculated aggregated behavior risk scores (ABRS) as described by Shahab et al.^9^. The CAM and CAM-MY scores were compared between the participants categorized based on ABRS (0–1 and 2–5), and no significant difference was noted (Fig. 4d). However, we observed that with increasing ABRS, participants' age also increased, while there was a significant decline in their CAM scores (Fig. 4e).

We also determined whether a particular health behavior characteristic will affect the score of the related question. For instance, we studied whether the recognition of smoking as a risk factor was different among smokers, non-smokers, and ex-smokers. We observed no significant difference in the average scores for the active and passive smoking-related questions among these groups (Supplementary Fig. S1a). We compared the Low PA CAM item responses between the participants categorized based on their PA levels—following PA guidelines or not following PA guidelines—and observed no significant difference between the average scores (Supplementary Fig. S1b). The responses for the Low FV question were also compared between the participants categorized based on their FV consumption levels—following or not following FV consumption guidelines—and no significant difference was observed between the average scores (Supplementary Fig. S1c). Moreover, the two BMI groups also attained similar scores on the body-weight related question (Supplementary Fig. S1d).

### Effect of formal years of education on CAM and CAM-MY scores

**Figure 5:**
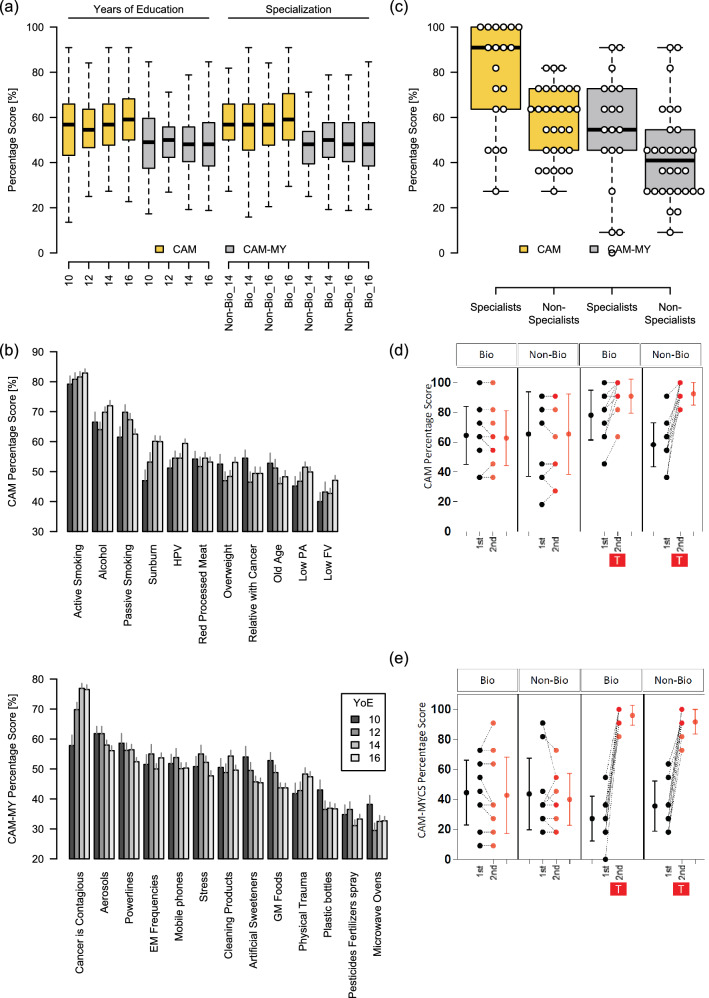
Effect of educational background on CAM and CAM-MY scores. (**a**) The study population was stratified according to the formal years of education (YoE) (10, 12, 14, or 16) or major Biology or Non-Biology at undergraduate (14 YoE) or masters (16 YoE) levels. Significance was determined by *ordinary one-way ANOVA.* Percentage scores of the study-participants for each (**b**) CAM and CAM-MY questions were compared between subgroups stratified according to the formal years of education. (**c**) CAM and CAM-MY scores were compared between cancer experts vs. non-specialists. Significance was determined by Significance was determined by *unpaired t-test.* The effects of formal cancer awareness training sessions on (**d**) CAM and (**e**) CAM-MY scores was determined. The study-participants (university students with biology or non-biology majors) were presented with the questions including CAM and CAM-MYCS items at baseline levels. After completing the baseline interview, the sample was randomized 1:1 to either the intervention or control group. The intervention group attended a brief training session (20 min). The control group did not receive any intervention. After 48 h the participants were presented with the same questions to assess the effect of training session. X- axis displays number of interviews (1st or 2nd) the symbol T in a red-box highlights the second interview that was conducted for the intervention group 48 h post-training.

A previous work associated the CAM and CAM-MY scores with post-16 years of qualification^11^. Here, we categorized the participants according to their formal years of education (YoE) (10, 12, 14, or 16 years). It was observed that the CAM and CAM-MY scores only slightly varied among these groups, and no significant difference was observed (Fig. 5a). The last two groups (14 and 16 YoE) were further classified into biology major and non-biology major groups. We observed that having a biology major does not significantly affect the CAM and CAM-MY scores in our population (Fig. 5a).

Next, we sought to determine the impact of formal education years on the correct recognition of CAM items as cancer risk factors. Active smoking was the most correctly recognized risk factor for all the four groups, with each group attaining an average score of ~ 80% for this CAM item (Fig. 5b, Supplementary Table 3). It was followed by alcohol consumption and passive smoking, for which each group attained an average score of > 61%. On the other hand, low FV, and low PA. remained the least recognized risk factors for all the four groups.

The responses to CAM-MY items also varied among the four education-based groups (Fig. 5b, Supplementary Table 4). However, this difference was most pronounced for the item "Cancer is contagious," with the average scores for this question, increasing with increasing years of education. However, the average scores for 14 and 16 YoE categories were nearly the same. For all the four groups, microwave ovens, fertilizers/pesticide sprays, and plastic bottles were the least recognized myths for all the groups, with the average scores of as low as ~ 30% for the related questions.

We also compared the correlation between CAM and CAM-MY scores separately for each of the education-based groups. A significant negative correlation was observed for each category. However, the strength of the negative association was significantly higher for 10 and 16 YoE (Supplementary Fig. S2).

We also compared the average CAM and CAM-MY scores between cancer-experts and non-medical students. The average CAM scores for cancer experts were higher than the non-experts (Fig. 5c). This indicates these measures successfully distinguish between groups known to have different levels of knowledge. However, the difference in the CAM-MY scores from the two groups was not significantly different (Fig. 5c).

### Effect of brief training sessions on CAM and CAM-MY scores

Next, we assessed the impact of brief training sessions on CAM and CAM-MY scores in students with biology or non-biology majors at the undergraduate level. We observed that in both groups, the CAM and CAM-MY scores were significantly improved after attending the training sessions (Fig. 5d,e). However, both groups benefited equally from these sessions, and no significant difference was noted between their post-training CAM and CAM-MY scores. The improvement in post-training CAM-MY scores was more pronounced than post-training CAM scores.

## Discussion

The present study explores the levels of cancer awareness among Pakistanis. To assess cancer awareness, we used two scales, i.e., Cancer Awareness Measure (CAM) and Cancer Awareness Measure–MYthical Causes Scale (CAM-MY). CAM is a validated, face-to-face questionnaire designed to measure the public's awareness of cancer symptoms and risk factors. On the other hand, CAM-MY is a more recently developed tool that assesses public beliefs in non-validated or the so-called mythical causes of cancer^11,12^. While CAM-MY items are carefully chosen based on scientific consensus, using reports from leading agencies^13^ and experts from various relevant disciplines, we cannot completely rule out causal relationships between the factors included within the tool and the development of cancer. Nevertheless, there is a possibility that future studies would provide convincing evidence on the causal relationship between either of these factors and carcinogenesis. For instance, there is some preliminary evidence of a weak association between certain forms of cancer and mobile phone use^14^. Hence, if the scientific consensus changes, the CAM-MY could be adapted accordingly.

In this study, we assessed the CAM and CAM-MY scores in the Pakistani population. We observed that people scored significantly better on the CAM scale in comparison to the CAM-MY scale. Interestingly, CAM scores were negatively associated with CAM-MY scores. Shahab et al. observed similar trends in the English population^9^. There is a possibility that some people merely believe that anything can cause cancer, and thus they endorsed both validated and non-validated risk-factors as cancer causes.

We also observed that years of formal education or a biology major at undergraduate or graduate level did not affect our population's cancer awareness levels. In contrast, Shahab et al.^9^ reported that having post-16 years of education is directly associated with cancer awareness levels in the English population. We can infer that the current Pakistani education system does not improve our understanding of cancer causes or risk factors over the general knowledge.

Shahab et al.^9^ have assessed the level of awareness on cancer risk factors and people's belief in non-validated causes of cancer using CAM and CAM-MY in the English population. They report that cancer awareness levels were associated with several socio-demographic factors, including age, social status, race, and education. Here, we observe that the major socio-demographic factor affecting the CAM scores was the participants' marital status, with the single participants scoring better at this scale. We hypothesized that in part this difference could be attributed to the participants' age, as married participants were mostly older. However, age displayed only a weak negative association with CAM scores. Hence, age could only be partially responsible for the difference between the CAM scores in married vs. single respondents.

Previous works have shown that a few health behavior characteristics are associated with improved cancer awareness. For instance, it has been shown that the participants that perform better on the CAM scale are more adherent to fruit and vegetable consumption^9^. The presented study found no association between most health behavior characteristics and CAM or CAM-MY scores. This is reassuring as it would suggest that lower cancer awareness levels do not necessarily result in an unhealthy lifestyle.

The medical professionals and oncologists scored lower on the CAM-MY scale in comparison to CAM scale. Their average percentage score on the CAM scale was above 90, while their average score on the CAM-MY scale was ~ 50 out of 100. Healthcare professionals have a hectic schedule with their long clinic hours and medical duties, and it becomes difficult for them to stay updated on the latest scientific literature. Generally, the public reaches out to the doctors and trust their perspective on healthcare-related issues. Hence, it is imperative to bridge the gap between basic sciences and clinical practice.

In the last decade, the misconceptions regarding cancer causes have massively increased^9^. This could be a reflection of changes in the way people access news^15^. It is an emerging trend for people who seek health information to use social media which is flooded with fake news and misinformation. Previous works have shown that belief in non-validated risk factors might be associated with increased anxiety, especially when they cannot control or modify the risk source^16,17^. Our study observed that most participants failed to identify the modifiable risk factors, including Low PA and Low FV. We suggest that by improving the knowledge of cancer causes and risk factors, we might also reduce fear and anxiety and make people feel more empowered about their ability to reduce their cancer risk. In addition, this study shows that people's understanding of cancer risk factors and myths could be markedly improved through brief training sessions. However, the awareness level may not be associated with cancer incidence. People are more aware of the carcinogenic effects of smoking because the tobacco companies are required by law to acknowledge the dangers of tobacco in their advertising campaigns and on cigarette boxes.

The CAM-MYCS was developed using iterative mixed-methods, it is possible that it does not reflect all common beliefs in mythical causes of cancer held by the public. Future studies are needed to develop cancer site-specific versions and explore variation in mythical beliefs between countries.

## Supplementary Information

Supplementary Information 1.

Supplementary Information 2.

## Data Availability

The datasets supporting the conclusions of this article are included within the article and supplementary files.
