# Supplementary material for: Cancer Awareness Measure (CAM) and Cancer Awareness Measure MYthical Causes Scale (CAM-MY) scores in Pakistani population
Source: Sci Rep. 2022 May 25;12:8887. doi: 10.1038/s41598-022-13012-8 (PMC9132919; doi:10.1038/s41598-022-13012-8)
Supplement: Supplementary file 1 — Supplementary Information 1. [file 41598_2022_13012_MOESM1_ESM.pptx]

## Slide 1
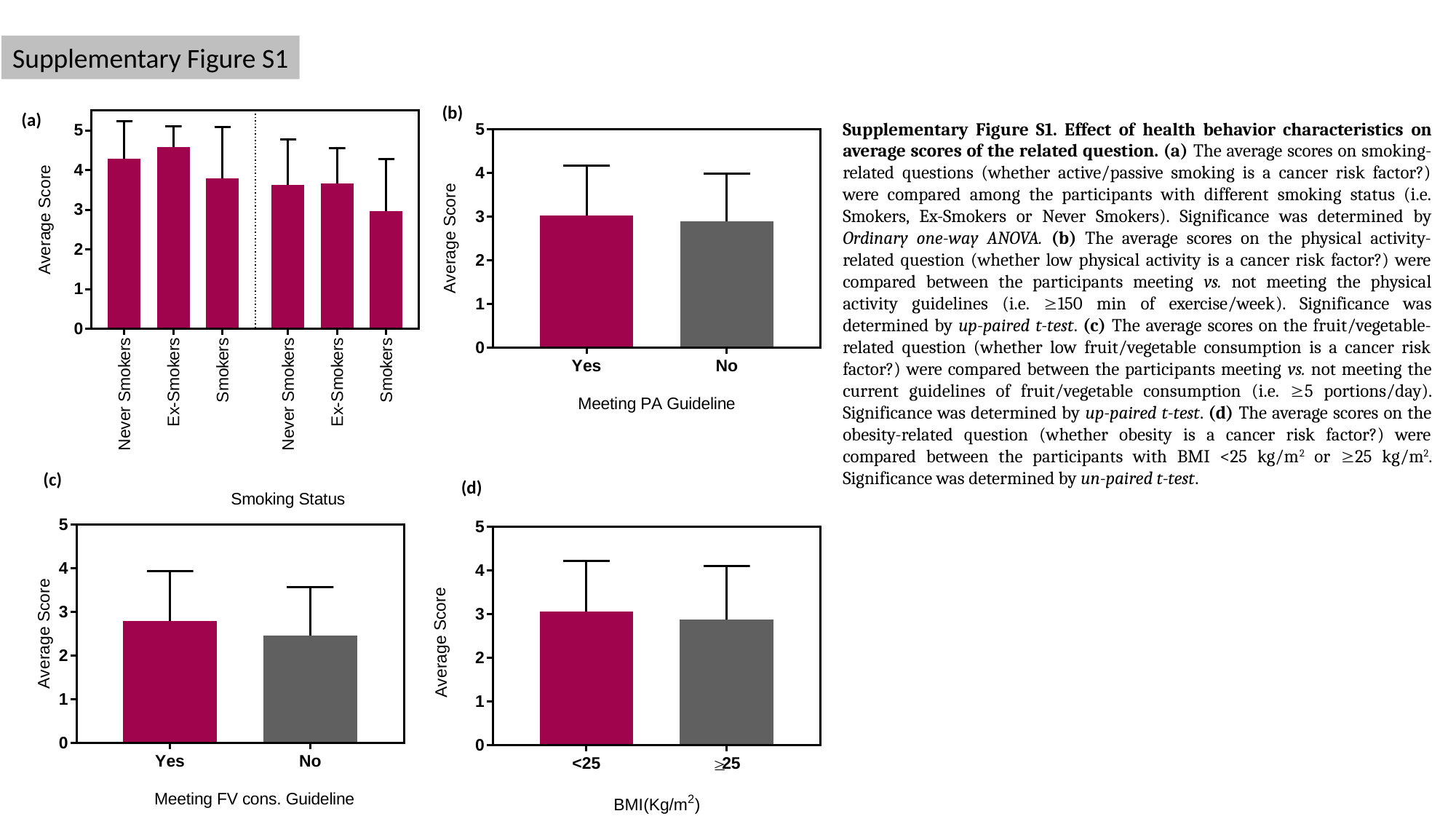

Supplementary Figure S1
(b)
(a)
Supplementary Figure S1. Effect of health behavior characteristics on average scores of the related question. (a) The average scores on smoking-related questions (whether active/passive smoking is a cancer risk factor?) were compared among the participants with different smoking status (i.e. Smokers, Ex-Smokers or Never Smokers). Significance was determined by Ordinary one-way ANOVA. (b) The average scores on the physical activity-related question (whether low physical activity is a cancer risk factor?) were compared between the participants meeting vs. not meeting the physical activity guidelines (i.e. ≥150 min of exercise/week). Significance was determined by up-paired t-test. (c) The average scores on the fruit/vegetable-related question (whether low fruit/vegetable consumption is a cancer risk factor?) were compared between the participants meeting vs. not meeting the current guidelines of fruit/vegetable consumption (i.e. ≥5 portions/day). Significance was determined by up-paired t-test. (d) The average scores on the obesity-related question (whether obesity is a cancer risk factor?) were compared between the participants with BMI <25 kg/m2 or ≥25 kg/m2. Significance was determined by un-paired t-test.
(c)
(d)

## Slide 2
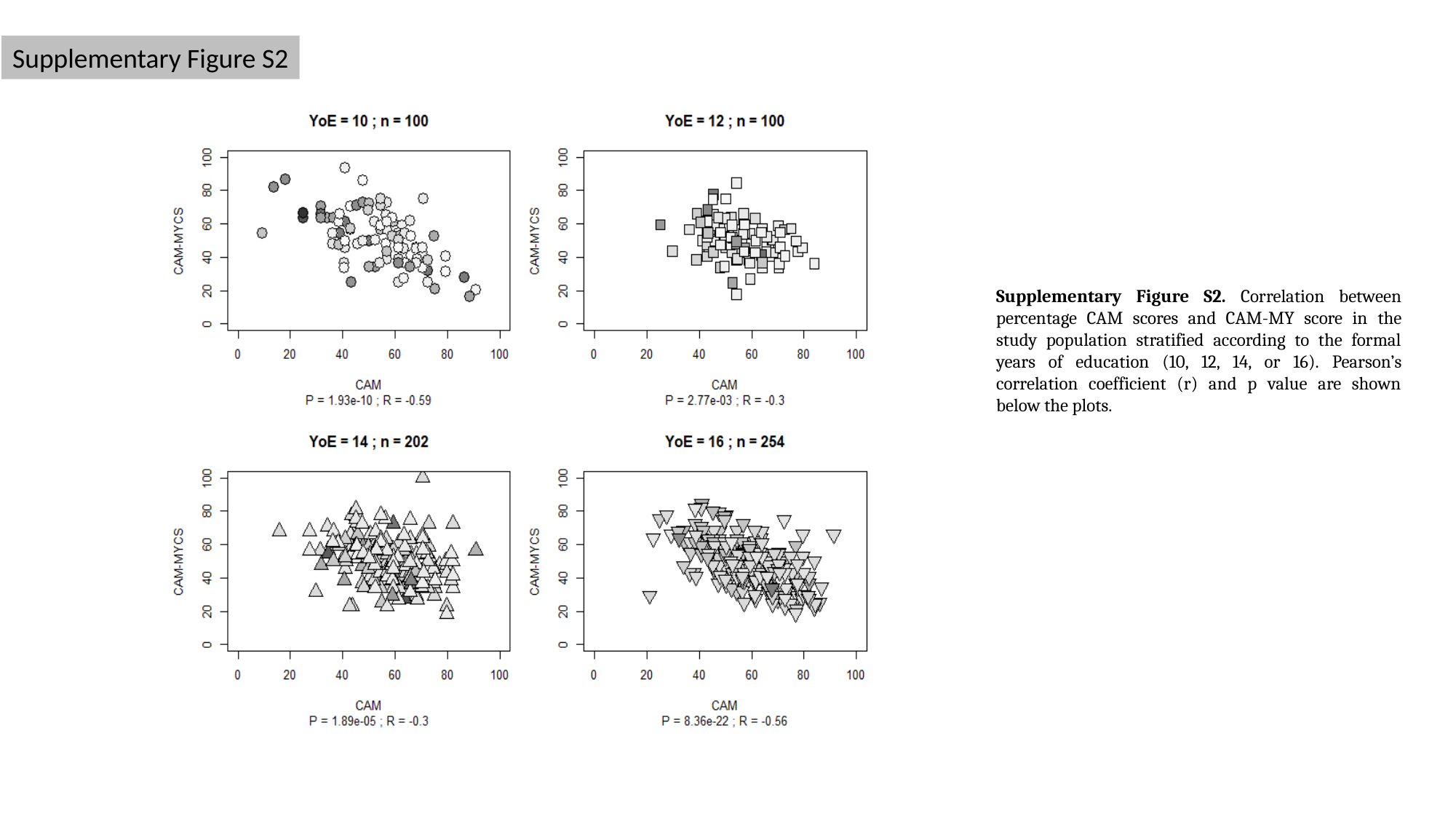

Supplementary Figure S2
Supplementary Figure S2. Correlation between percentage CAM scores and CAM-MY score in the study population stratified according to the formal years of education (10, 12, 14, or 16). Pearson’s correlation coefficient (r) and p value are shown below the plots.
