# Supplementary material for: Cancer Awareness Measure (CAM) and Cancer Awareness Measure MYthical Causes Scale (CAM-MY) scores in Pakistani population
Source: Sci Rep. 2022 May 25;12:8887. doi: 10.1038/s41598-022-13012-8 (PMC9132919; doi:10.1038/s41598-022-13012-8)
Supplement: Supplementary file 2 — Supplementary Information 2. [file 41598_2022_13012_MOESM2_ESM.docx]

| **Supplementary Table 1: Average scores for CAM items.** | | | | | | | | | | | |
| --- | --- | --- | --- | --- | --- | --- | --- | --- | --- | --- | --- |
|  | Low FV | Low PA | Old Age | HPV | Overweight | Relative with Cancer | Red Processed Meat | Sunburn | Passive Smoking | Alcohol | Active Smoking |
| Strongly Disagree | 4.1 | 5.9 | 8.2 | 5.5 | 9.6 | 9.1 | 6.1 | 12.7 | 22 | 27.9 | 47.9 |
| Disagree | 30.2 | 35.4 | 33.7 | 36.9 | 33.1 | 34.8 | 38.7 | 44.2 | 42.7 | 41.8 | 42.1 |
| Neutral | 17.4 | 17.1 | 13.7 | 38.3 | 15.2 | 12.2 | 24.2 | 10.8 | 13.7 | 14 | 2 |
| Agree | 34.6 | 32.5 | 33.5 | 14.5 | 34.3 | 33.7 | 25.2 | 23.3 | 16.5 | 12.2 | 4.7 |
| Strongly Agree | 13.7 | 9 | 10.8 | 4.9 | 7.8 | 10.2 | 5.8 | 9 | 5.2 | 4.1 | 3.4 |

| **Supplementary Table 2: Average scores for CAM-MY items.** | | | | | | | | | | | | | |
| --- | --- | --- | --- | --- | --- | --- | --- | --- | --- | --- | --- | --- | --- |
|  | Pesticides  Fertilizers spray | Microwave  Ovens | Plastic bottles | GM Foods | Artificial  Sweeteners | Physical  Trauma | Cleaning  Products | Mobile  phones | Stress | EM  Frequencies | Power lines | Aerosols | Cancer is Contagious |
| Strongly Agree | 19.7 | 18.4 | 15.5 | 6.1 | 4.7 | 9.3 | 5.5 | 5.6 | 8.1 | 4.7 | 4 | 2.7 | 4.4 |
| Agree | 47.4 | 54 | 47.3 | 34.5 | 40.4 | 38.7 | 32.2 | 35.8 | 32.9 | 34.5 | 27.7 | 22 | 12.7 |
| Neutral | 15.9 | 9 | 13.3 | 34.5 | 20.4 | 16.6 | 22.6 | 18 | 15.5 | 16.9 | 22.7 | 24.1 | 5.6 |
| Disagree | 14.2 | 14.3 | 18.8 | 20 | 29.1 | 28.7 | 32.2 | 30 | 34.5 | 34.1 | 34.3 | 41.3 | 42.2 |
| Strongly Disagree | 2.9 | 4.3 | 5.2 | 5 | 5.2 | 6.6 | 7.6 | 10.5 | 8.7 | 9.8 | 11 | 9.9 | 35.1 |

| **Supplementary Table 3: Effects of Formal Years of Education (YoE) on the average score for each CAM question.** | | | | | | | | | | | |
| --- | --- | --- | --- | --- | --- | --- | --- | --- | --- | --- | --- |
| YoE | Active Smoking | Passive Smoking | Alcohol | Low FV | Red Processed Meat | Overweight | Sunburn | Old Age | Relative with Cancer | HPV | Low PA |
| 10 | 79.2 | 61.5 | 66.5 | 40 | 54.2 | 52.5 | 47 | 52.8 | 54.5 | 51.2 | 45.2 |
| 12 | 80.8 | 69.8 | 64 | 43.2 | 51.7 | 47 | 53.2 | 51.2 | 46.5 | 54.5 | 46.8 |
| 14 | 81.6 | 67.3 | 69.8 | 42.7 | 54.5 | 48.4 | 60.1 | 46 | 49.4 | 54.5 | 51.5 |
| 16 | 82.9 | 62.5 | 72 | 47.1 | 53.2 | 53.1 | 60 | 48.3 | 49.4 | 59.4 | 49.9 |

| **Supplementary Table 4: Effects of Formal Years of Education (YoE) on the average score for each CAM-MY question.** | | | | | | | | | | | | | |
| --- | --- | --- | --- | --- | --- | --- | --- | --- | --- | --- | --- | --- | --- |
| YoE | GM  Foods | Cancer  is Contagious | Microwave  Ovens | Aerosols | Plastic  bottles | Mobile  phones | Cleaning  Products | Artificial  Sweeteners | Power lines | Stress | Physical  Trauma | EM  Frequencies | Pesticides  Fertilizers  spray |
| 10 | 52.8 | 57.8 | 38.2 | 61.8 | 43 | 51.8 | 50.5 | 54 | 58.6 | 50.8 | 41.8 | 51.5 | 34.8 |
| 12 | 48.8 | 69.8 | 29.5 | 61.8 | 36.5 | 53.8 | 48.8 | 49.5 | 56.2 | 55 | 42.8 | 55 | 36.5 |
| 14 | 43.7 | 76.9 | 32.5 | 58 | 36.9 | 50.1 | 54.3 | 45.7 | 56.4 | 52.2 | 48.3 | 50 | 31.1 |
| 16 | 43.7 | 76.5 | 32.7 | 56.1 | 36.7 | 50.3 | 49.6 | 45.4 | 52.4 | 47.7 | 47.4 | 53.7 | 33.3 |
